# Molecular characterization of *MET* fusions from a large real‐world Chinese population: A multicenter study

**DOI:** 10.1002/cam4.6047

**Published:** 2023-06-16

**Authors:** Hui Xia, Junhua Zhang, Tong Chen, Mingzhao Wang, Dongna Chen, Tongguo Si, Yutao Liu

**Affiliations:** ^1^ Thoracic Surgery Department The Fourth Medical Center of PLA General Hospital Beijing China; ^2^ Department of Radiation Oncology Fudan University Shanghai Cancer Center Shanghai China; ^3^ Department of Oncology Shanghai Medical College, Fudan University Shanghai China; ^4^ Shanghai Clinical Research Center for Radiation Oncology Shanghai Key Laboratory of Radiation Oncology Shanghai China; ^5^ Department of Medical Oncology National Cancer Center/National Clinical Research Center for Cancer/Cancer Hospital, Chinese Academy of Medical Sciences & Peking Union Medical College Beijing China; ^6^ Department of Medical Oncology Sanhuan Cancer Hospital of Chaoyang District Beijing China; ^7^ Department of Interventional Treatment Tianjin Medical University Cancer Hospital and Institute Tianjin China

**Keywords:** *KIF5B‐MET*, lung cancer, *MET* fusion, novel partners, prevalence

## Abstract

**Purpose:**

*MET* is a notable driver gene in the diversity of aberrations with clinical relevance, including exon 14 skipping, copy number gain, point mutations, and gene fusions. Compared with the former two, *MET* fusions are severely under‐reported, leaving a series of unanswered questions. In this study, we addressed this gap by characterizing *MET* fusions in a large, real‐world Chinese cancer population.

**Methods:**

We retrospectively included patients with solid tumors who had DNA‐based genome profiles acquired through targeted sequencing from August 2015 to May 2021. *MET* fusion‐positive (*MET*+) patients were subsequently selected for clinical and molecular characterization.

**Results:**

We screened 79,803 patients across 27 tumor types and detected 155 putative *MET* fusions from 122 patients, resulting in an overall prevalence of 0.15%. Lung cancer comprised the majority of *MET*+ patients (92, 75.4%). Prevalence was markedly higher in liver cancer, biliary tract cancer, and renal cancer (range 0.52%–0.60%). It was lower in ovarian cancer (0.06%). A substantial proportion (48/58, 82.8%) of unique partners were reported for the first time. High heterogeneity was observed for partners, with *ST7*, *HLA‐DRB1*, and *KIF5B* as the three most common partners. Mutational landscape analysis of lung adenocarcinoma (*n* = 32) revealed a high prevalence of *TP53* in *MET*+ alterations, *EGFR* L858R, *EGFR* L861Q, and *MET* amplification.

**Conclusion:**

To our knowledge, this is currently the largest study in characterizing *MET* fusions. Our findings warrant that further clinical validation and mechanistic study may translate into therapeutic avenues for *MET*+ cancer patients.

## 1 INTRODUCTION

*MET* as an oncogenic driver in lung cancer (LC) has been reported in a number of other malignancies, including gastric cancer, hepatocellular carcinoma, papillary renal cell carcinoma, thyroid cancer, glioma, and sarcoma.^1^, ^2^, ^3^, ^4^ Compared with other driver genes, *MET* is notable in the diversity of its aberrations with established clinical relevance, including exon 14 skipping, copy number gain (CNG), point mutations, and gene fusions.^1^, ^4^, ^5^ The *MET* gene is located on chromosome 7q21‐23 and encodes for a receptor tyrosine kinase, which homodimerizes upon binding to hepatocyte growth factor (HGF) and activates downstream signaling pathways such as RAS/ERK/MAPK and PI3K/AKT, thereby promoting tumorigenesis and tumor progression.^6^ Normally, activated MET can be autoregulated by transphosphorylation‐mediated recruitment of CBL E3 ligase, which then mediates MET degradation and thereby establishes a negative feedback loop. However, cells may undergo *MET*‐driven oncogenesis or acquisition of therapeutic resistance in the case of elevated MET signaling through gene amplification, constitutive activation or expression via gene fusions, and/or loss of the transphosphorylation site through exon 14 skipping or point mutations.^1^

*MET* aberrations are currently most extensively characterized in LC, which shows a prevalence of approximately 5%,^5^ including exon 14 skipping^7^, ^8^, ^9^ and CNG.^4^, ^10^, ^11^, ^12^ In comparison, there is a paucity of data on *MET* fusions. *MET* fusions are clinically relevant in that it results in constitutive MET activation, thereby promoting oncogenesis or therapeutic resistance.^1^ Apart from isolated reports of response to MET tyrosine kinase inhibitors (TKIs)^13^, ^14^, ^15^ or mechanism of acquired resistance to EGFR‐TKIs,^16^ current knowledge regarding *MET* fusions is mostly derived from three studies, which identified 2 (0.59%, 2/337) *MET* fusions in lung adenocarcinoma,^17^ 1 (0.04%, 1/2410)^18^ and 15 (0.26%, 15/5695) fusions involving the MET kinase domain (KD) in non‐small cell lung cancer (NSCLC), respectively.^19^ The low prevalence and heterogeneity in partner genes and breakpoints warrant the screening of large cohorts for characterizing *MET* fusions.

Herein, we report a retrospective study that screened 79,803 patients and identified 155 putative *MET* fusions, which involved 58 unique partners, 48 of which reported for the first time. Subsequent characterization revealed the genomic landscape of these fusions and *MET* fusion‐positive lung adenocarcinoma cases. We also highlight the therapeutic relevance of *MET* fusions for targeted therapy with select LC cases.

## 2 PATIENTS AND METHODS

### 2.1 Patient information and samples

This study screened the genomic profiles of patients with solid tumors for *MET* fusions and analyzed the clinical and molecular characteristics of the *MET* fusion‐positive patients. The study was approved by the institutional review board of Tianjin Medical University Cancer Hospital and Institute. Patients with solid tumors who had available genome profiles acquired through targeted sequencing from August 2015 to May 2021 were retrospectively included. There was no preselection based on tumor type, histologic subtype, age, sex, clinical stage, or sample type. A total of 79,803 patients were screened, corresponding to 41,404 tumor tissue samples, 35,770 plasma, 2,242 pleural effusion, and 387 cerebrospinal fluid. In this work, only one sample of each patient was included. After we reviewed the sample records, several patients had two or more types of samples or had more than two specimens with the same sample type (such as plasma, tissue). For those who had two or more types of samples, only the tissue sample was included. For those who had more than two specimens with the same sample type, only the sample detected the first time was included. Genomic profiling was performed with hybrid capture‐based next‐generation sequencing (NGS) using panels targeting 8, 41, 68, 108, 168, or 520 cancer‐related genes (Burning Rock Biotech). These panels were designed and validated for the identification of base substitutions, insertions, deletions, copy number variations (CNV), and gene fusions.^20^

### 2.2 DNA extraction, library construction, targeted sequencing, and bioinformatic analysis

All wet‐lab procedures were performed at a College of American Pathologists (CAP)‐ and Clinical Laboratory Improvement Amendments (CLIA)‐certified clinical diagnostic laboratory (Burning Rock Biotech). Briefly, DNA from tumor tissues was extracted with a QIAamp DNA tissue kit, and cell‐free DNA from liquid biopsies was extracted with a QIAamp Circulating Nucleic Acid kit (Qiagen). DNA library construction and targeted sequencing with a commercial panel of 8, 41, 68, 108, 168, or 520 genes (Burning Rock Biotech) were performed as previously described.^21^, ^22^ Sequencing was conducted on Nextseq500 (Illumina) at a target depth of 1000× for tumor samples and 10,000× for liquid biopsy samples.

### 2.3 NGS data analysis

All the reads were mapped to the human genome (hg19) with Burrows–Wheeler Aligner (BWA).^23^ Local alignment optimization, mark duplication, and variant calling were performed using Genome Analysis ToolKit (GATK),^24^ picards, and VarScan.^25^ Gene rearrangements were called with FACTERA,^26^ and copy CNVs were analyzed based on sequencing depth. Variants were filtered using the VarScan fpfilter pipeline, and loci with depths <100 were filtered out. At least two and five supporting reads were needed for insertions and deletions (indels), while eight supporting reads were needed to call SNVs, in both plasma and tissue samples. According to the ExAC, 1000 Genomes, dbSNP, ESP6500SI‐V2 database, variants with population frequency over 0.1% were grouped as SNP and excluded from further analysis. The remaining variants were annotated with ANNOVAR^27^ and SnpEff.^28^ Tumor mutation burden (TMB) was calculated as number of nonsynonymous somatic alterations on the coding regions of the targeted genes per million base pairs after excluding alterations with an allelic frequency of <2% from tissue samples or <0.2% from liquid biopsy samples. The concurrent alterations with *MET* fusions were investigated in 27 *MET* fusion‐positive lung adenocarcinoma (LUAD) patients who underwent targeted sequencing using a 168‐ or 520‐gene panel. In addition, the concurrent lung cancer driver alterations with *MET* fusions were also investigated in 32 *MET*+ LUAD patients who underwent targeted sequencing using panels targeting 8, 41, 68, 108, 168, or 520 cancer‐related genes. These panels included eight identical genes, also known as classic lung cancer oncogenes, including *EGFR*, *KRAS*, *BRAF*, *ALK*, *ROS1*, *MET*, *RET*, and *ERBB2*.

### 2.4 Statistical analysis

All statistical analyses were performed using the R programming language. Chi‐square or Fisher's exact test was used to compare between two groups in the proportions of values of a nominal variable, such as percentage of *MET* fusion‐positive patients. Wilcoxon signed‐rank test was used to compare the central tendency of a continuous variable, such as TMB, between two groups. All tests were two‐sided with a significance level set at *p* < 0.05.

## 3 RESULTS

### 3.1 Patient characteristics

In this study, we retrospectively screened 79,803 patients with solid tumors who had DNA‐based genomic profiles acquired with panel‐based targeted sequencing. A total of 155 putative *MET* fusions events were identified in 122 patients (151 samples), resulting in an overall prevalence of 0.15% (122/79803).

**FIGURE 1:**
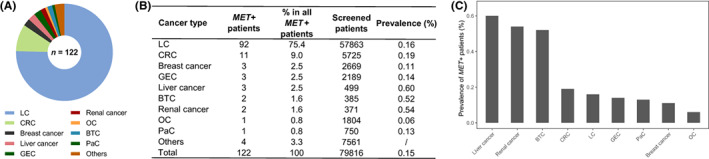
Distribution and prevalence of *MET* fusions identified in this study. (A) Distribution of *MET* fusions across cancer types. (B) Numbers of screened and *MET* fusion‐positive patients per cancer type. (C) Tumor type‐specific prevalence rates of *MET* fusion‐positive patients sorted by level. BTC, biliary tract cancer; CRC, colorectal cancer; GEC, gastroesophageal cancer; LC, lung cancer; *MET*+, *MET* fusion‐positive; OC, ovarian cancer; PaC, pancreatic cancer.

Figure 1 shows the characteristics of *MET* fusions (hereafter referred to as *MET*+) across cancer types. LC represents the largest subset (*n* = 92, 75.4%), followed by colorectal cancer (CRC, *n* = 11, 9.0%). These two cancer types showed similar prevalence rates of *MET* fusions (0.16% and 0.19%), which were comparable with overall prevalence. Although liver cancer (*n* = 3), biliary tract cancer (BTC, *n* = 2), and renal cancer (*n* = 2) patients showed higher prevalence ranging from 0.52% to 0.60%, ovarian cancer (OC, *n* = 1) showed a markedly lower rate at 0.06%, these results may be artifacts due to the small number of *MET*+ patients. Pairwise comparison between different tumor types revealed no significant difference in prevalence levels (Figure S1). Other clinicopathologic characteristics of the patients are summarized in Table 1. Sixty‐three (51.6%) of the *MET*+ patients were men and 53 (43.4%) were women. Gender information was not documented for the remaining six cases (*n* = 1.2%). Median age was 58 years with a range of 27–87 years. More than half had stage III disease (*n* = 65, 53.3%), followed by stage IV disease (*n* = 41, 33.6%).

**TABLE 1:** Summary of clinicopathological characteristics of all *MET* fusion‐positive patients and those with lung adenocarcinoma.

Feature	All *MET + n* (%)	*MET+* LUAD *n* (%)
Number of patients	122	32
Age, in yrs, median (range)	58 (27–87)	58 (40–79)
Sex		
Female	53 (43.4)	15 (46.9)
Male	63 (51.6)	15 (46.9)
NA	6 (4.9)	2 (6.3)
Stage		
I	3 (2.5)	0
II	5 (4.1)	3 (9.4)
III	65 (53.3)	12 (37.5)
IV	41 (33.6)	17 (53.1)
NA	8 (6.6)	0
Number of targeted genes		
8	10 (3.3)	4 (12.5)
41	2 (1.6)	0
68	4 (3.3)	1 (3.1)
108	4 (3.3)	0
168	46 (37.7)	14 (43.8)
520	56 (45.9)	13 (40.6)

Abbreviations: LUAD, lung adenocarcinoma; *MET*+, *MET* fusion‐positive; NA, not available.

### 3.2 Molecular characteristics of *MET* fusions

**FIGURE 2:**
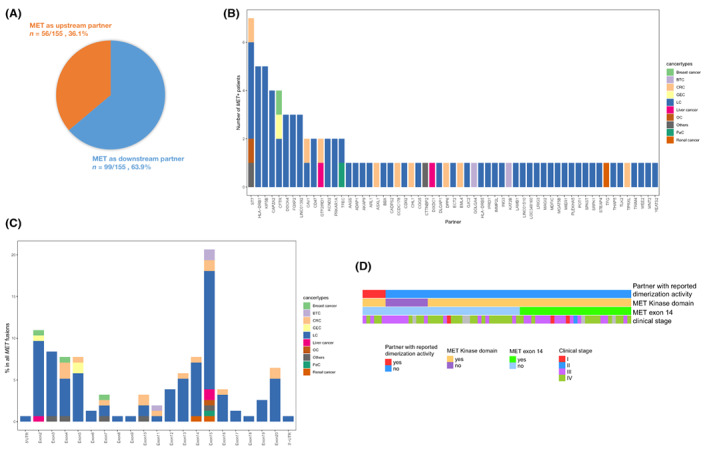
Characterization of all 155 *MET* fusions identified in this study. (A) Distribution of *MET* as the 5' or 3' partner. (B) Distribution of the partners among nonintergenic fusions. (C) Distribution of breakpoints at the specific region of *MET*. (D) Details of clinical stage and inclusion of exon 14 or kinase domain corresponding to each fusion with *MET* as the 3' partner. BTC, biliary tract cancer; CRC, colorectal cancer; GEC, gastroesophageal cancer; LC, lung cancer; *MET*+, *MET* fusion‐positive; OC, ovarian cancer; PaC, pancreatic cancer; UTR, untranslated region.

A by‐patient view of clinical and molecular characteristics for each *MET*+ patient is provided in Table S1. Most (87/122, 71.3%) patients harbored ≥1 fusion in which *MET* was the 3' partner, among whom 21 (17.2%) carried multiple *MET* fusions, including 12 (9.8%) cases in which *MET* was also found as a 5' partner, and the remaining 9 patients had >1 (range 2–5) fusions, all of which with *MET* as the 3' partners. In terms of fusion events, *MET* was the 3' partner in 99 (63.9%) events and the 5' partner in the remaining 56 (36.1%; Figure 2A).

The majority (48/58, 82.8%) of the *MET* partners identified in this study, to our knowledge, has not been previously reported.^1^ As summarized in Figure 2B, in addition to LC (*n* = 47), these novel fusions were also identified in CRC (*n* = 7), BTC (*n* = 2), liver cancer (*n* = 2), gastroesophageal cancer (GEC, *n* = 1), breast cancer (*n* = 1), pancreatic cancer (1 event), and other cancers (*n* = 1). A considerable proportion of *MET* fusions were intergenic (65/155, 41.9%). Precise 59 partners were found in nonintergenic fusions, with 76.3% of partners (*n* = 45) identified only once and 86.4% of partners (*n* = 51) identified ≤2 times. *ST7* stood out as the most common partner occurring in 7 events, followed by *HLA‐DRB1* and *KIF5B* (5 events each), and *CAPZA2* and *CFTR* (4 events each). Notably, all nonintergenic fusions with *HLA‐DRB* or *CAPZA2* had *MET* at the 3' end and occurred in LC. Fusions with *KIF5B* were also all detected in LC and had *MET* as the 3' partner except for one occasion. Besides LC (4 events), *ST7‐MET* was also present in CRC, OC, and other cancer types (1 event for each). Interestingly, apart from LC (2 events), fusions with *CFTR* were detected in 1 GEC and 1 breast cancer case in the forms of *MET‐CFTR* and *CFTR‐MET*, respectively. Analysis of breakpoint distribution indicated exon 15 as the most common exon (Figure 2C). Figure 2D shows a by‐patient view of clinical stages and fusions‐specific features of the 70 nonintergenic fusions with *MET* as the 3' partner. Fewer than half of them (29/70, 41.4%) harbored *MET* exon 14 while most (59/70, 84.3%) had intact encoding sequences for MET KD. In addition, we found that 41.7% (50/122) of *MET*+ patients harbored concurrent *MET* amplification. The details are summarized in Table S2.

### 3.3 Mutational landscape of *MET* fusions in lung adenocarcinoma

**FIGURE 3:**
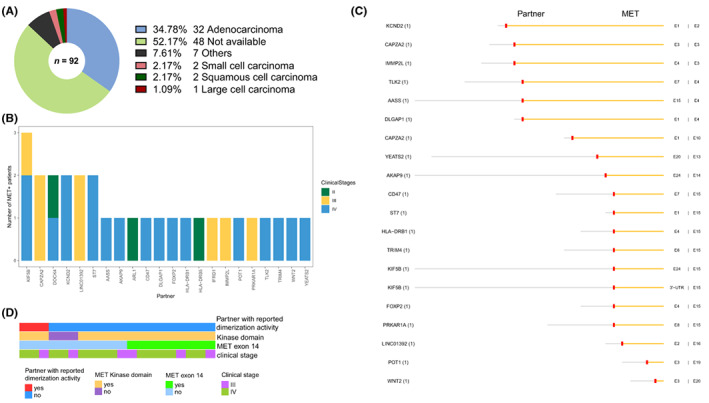
Characterization of the 54 *MET* fusions detected in lung adenocarcinoma patients. (A) Distribution of histologic subtypes among the 92 lung cancers carrying *MET* fusions. (B) Distribution of the partners among nonintergenic fusions. (C) Exon makeup of each of the 20 nonintergenic fusions with *MET* at the 3' end. (D) Details of clinical stage and inclusion of exon 14 or kinase domain corresponding to each fusion with *MET* as the 3' partner.

LUAD was the predominant histologic subtype with *MET* fusions, we then focused on the 32 *MET*+ LUAD cases for further characterization (Figure 3A). These patients showed a median age of 58 years (range 40–79) and equal numbers of men and women (15/32, 46.9% for each; Table 1). Similar to all *MET*+ cases, most (29/32, 90.6%) had stage III or IV disease.

These patients collectively carried 54 fusions with 22 unique partners. A total of 13 patients carried multiple (range 2–5) fusions. Excluding 25 intergenic ones, the remaining corresponded to 22 partners, including 15 novel ones. Most partners occurred only once except for *KIF5B*, *CAPZA2*, *DOCK4*, *KCND2*, *LINC01392*, and *ST7* (Figure 3B). Figure 3C illustrates the 20 nonintergenic fusions with *MET* as the 3' partner, of which the majority (17/20, 85.0%) harbored MET KD or lacked exon 14 (11/20, 65.0%; Figure 3D).

**FIGURE 4:**
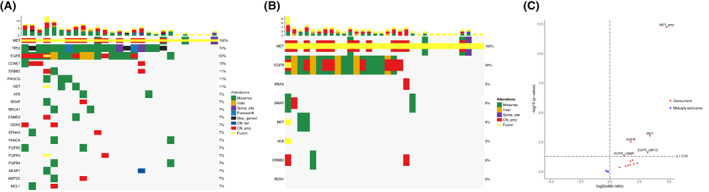
Mutational landscape of *MET* fusion‐positive lung adenocarcinoma (LUAD). (A) An oncoprint showing the 20 most frequently altered genes. (B) An overview of aberrations in eight classic driver genes in lung cancer. (C) Concurrent alterations in *MET* fusion‐positive LUAD. Concurrence was defined as an occurrence in *MET* fusion‐positive LUAD with an odds ratio >1 compared with *MET* fusion‐negative LUAD with a *p*‐value of <0.05. EGFR, *EGFR* alterations other than L858R or L861Q mutations. MET, *MET* alterations other than fusions or amplifications. Indel, insertion and deletion; CN, copy number.

Analysis of the mutational landscape was then performed using the 27 genome profiles acquired with a 168‐ or 520‐gene panel (Table 1). As shown in Figure 4A, aberrant *TP53* (74%) and *EGFR* (63%) had high frequencies, while other drivers, such as *ERBB2* (11%), *RET* (11%), and *BRAF* (7%), had relatively lower alteration rates. The most common concurrent *MET* alteration was *MET* amplification (12/27, 44.4%). *TP53* aberrations were predominantly point mutations, including 13 (48.1%) missense, 3 (11.1%) nonsense, and 2 (7.4%) frameshift mutations. *EGFR* alterations included equal numbers of missense mutations and amplification (7 for each) as well as 3 indels and 1 fusion. The alteration frequencies of 8 LC‐associated driver genes were investigated in 32 *MET*+ LUAD patients who underwent a panel of 8, 41, 68, 108, 168, or 520 genes. *KRAS* (3%), *ALK* (3%), and *ROS1* (0%) presented with low alteration frequencies (Figure 4B). Consistently, Fisher's exact test identified that *MET* amplification, *EGFR* L858R, or *EGFR* L861Q, and *EGFR* alterations significantly co‐occur with *MET* fusions in LUAD (Figure 4C).

### 3.4 Case vignettes

Next, we describe three cases that highlight the diverse therapeutic implications of different *MET* fusions. The first was patient P69, a 67‐year‐old woman who underwent multiple lines of treatment for recurrent stage IV LC after 3 years of surgery (Figure 3B). She began fifth‐line crizotinib treatment due to the presence of *HLA‐DRB1‐MET* (H4:M15) and achieved rapid partial response (PR) but developed brain metastasis 1 year later. Afterward, cabozantinib was started but the patient manifested intracranial progression within 1 month. She then received alectinib and crizotinib plus anlotinib. The patient achieved respective progression‐free survival (PFS) of 3 and 7 months. Genomic profiling 5 months later identified acquired *MET* Y1230C and Y1230H mutations in addition to *HLA–DRB1–MET* fusion, suggesting one or both point mutations as a potential mechanism of acquired resistance to crizotinib plus anlotinib.

The second patient was a 49‐year‐old woman (P13) with stage III LUAD positive for *EGFR* L858R mutation (abundance: 1.9%). Plasma‐based genome profiling also identified low‐grade *MET* amplification (copy number: 2.4) and *LINC01392‐MET* (L2:M15) when the patient was on fourth‐line treatment with osimertinib plus anlotinib (Figure 3A). She received osimertinib plus crizotinib upon progression 5 months after the profiling. Two months later, *MET* amplification was undetectable, whereas the *LINC01392‐MET* persisted in as similar abundance, along with *EGFR* L858R with drastically increased abundance (72.2%) and newly emerged *IMMP2L‐MET* (I4:M3) at low abundance. The patient progressed another 3 months later with the best response of PR. Whether one or both of the fusions were resistant to crizotinib and contribute to disease progression was unclear.

In the third highlighted case, a 62‐year‐old woman (P107) had a *KIF5B‐MET* (K24:M15)‐positive LUAD resistant to second‐line crizotinib, as indicated by rapid progression within 2 months, which contrasted with previous report of durable response (10 months) to crizotinib in a patient with stage IV LUAD harboring *KIF5B‐MET* (K24:M14). Together, these cases demonstrated heterogeneity in the response to multi‐kinase and selective MET‐TKIs among carriers of different *MET* fusions.

## 4 DISCUSSION

Although *MET* was first identified as part of an oncogenic gene arrangement with *TRP*,^29^ the prevalence of its fusions is far lower than that of exon 14 skipping mutations or amplification. As a result, *MET* fusions and their therapeutic implications have been largely ignored. In this study, we addressed this challenge with a large‐scale screening of 79,803 patients across 27 tumor types, which led to the detection of 155 putative *MET* fusions from DNA‐based genomic profiles of 122 patients. Subsequent analyses answered fundamental questions such as prevalence rates in different tumor types, whether the fusions were enriched in any of these tumors, percentage of *MET* as the 3' partner and carriers of multiple fusions, location of the *MET* sequence, and breakpoints and partners. Notably, this study also identified 48 novel partners, which provided candidates for preclinical research into their biology and potential as drug targets. In terms of cancer types, LC patients represented the largest *MET*+ population (92/122, 75.4%) and LUAD as the largest histologic subtype (32/122, 26.2%). Analysis of the mutational landscape of *MET*+ LUAD revealed a prevalence of altered *TP53* or *EGFR* much higher than those of other genes and found *MET* amplification, *EGFR* L858R, and *EGFR* L861Q as frequently concurrent aberrations.

*MET* amplification and exon 14 skipping alterations are well‐known oncogenic drivers in multiple cancer types. A number of previous studies on the prevalence and clinicopathological characteristics of *MET* amplification/exon 14 skipping alterations in solid cancers across countries have been documented.^9^, ^30^, ^31^, ^32^, ^33^ However, *MET* fusions are poorly defined. To our knowledge, studies on *MET* fusions in large cohorts of tumors are scarce. Pekova et al. have reported *EML4‐MET* occuring in a pediatric papillary thyroid carcinoma patient (PTC, 1.08%, 1/93) from a large cohort.^34^ Although pediatric PTC patients were not included in this work, *EML4‐MET* was detected in a stage IV CRC patient (0.02%, 1/5725). A recent study by Yang et al. has revealed *MET* fusions in solid tumors from a multicenter study in China, indicating its incidence with 0.34% (37/10882) across cancer types and 0.07% (4/5835) in LC,^35^ which were 0.15% (122/79803) and 0.16% (92/57863) in the present study. Of three *MET* fusions, including *COMETT (LINC01510)‐MET*, *PRKAR1A‐MET*, and *SEMA3D‐MET*, detected in LC and validated by RNA‐sequencing in Yang's work,^35^ two *MET* fusions (*COMETT (LINC01510)‐MET* and *PRKAR1A‐MET*) were also detected in LC in the present work.

This study provided a comprehensive account of the frequencies of *MET* fusions in various cancer types. Prevalence was 0.15% overall and ranged from 0.06% to 1.38% across tumor types. LC, CRC, breast cancer, and GEC each had more than 2,000 screened patients and showed similar, relatively low rates. In contrast, BTC, liver cancer, and renal cancer, all with <500 screened patients, showed markedly higher rates, which may or may not be an artifact due to the small sample size and warrants further investigation. Pairwise Fisher's exact test showed no significant difference among the tumor‐specific prevalence rates (Figure S1). Interestingly, although there is evidence of *MET* fusions enriched in gliomas, including one report of ~10% prevalence in pediatric glioblastoma^36^ and one of 214 fusions detected from 272 glioma samples with RNA‐seq,^37^ none of the 66 screened gliomas patients in this study was *MET*+. This discrepancy was likely attributable to the different patient demographics and method of genome profiling, which suggested the significance of combining DNA‐ and RNA‐based detection in future studies of *MET* rearrangements.

Our analysis revealed substantial heterogeneity in *MET* fusion partners, which was distinct from other oncogenic driver fusions such as those involving *ALK* ^38^ or *RET*.^20^ Among the 58 unique partners, 44 were identified only once and 50 no more than twice. The top 3 most common partners *ST7*, *HLA‐DRB1*, and *KIF5B* were detected in 11.0% (17/155) events. In stark contrast, in a study of NSCLC patients confirmed to be *ALK*‐positive via immunohistochemistry and fluorescence in situ hybridization, those carrying fusions with one of the three most common partners (*EML4*, *KIF5B*, and *HIP1*) constituted 86.1% of (136/158) cases.^38^ A study screening DNA‐based genomic profiles for *RET*‐positive cancer patients showed that 74% of fusions involved *KIF5B*, *CCDC6*, or *NCOA4*.^20^ In comparison, *MET* was similar to *NTRK* in that both fusions were rare (*NTRK*: 899/295675, 0.30%) and demonstrated no marked partner preference. In a similar study of *NTRK* fusions in cancer patients, those harboring fusions with any of the 3 most frequent partners *ETV6*, *TPM3*, and *LMNA* comprised 22.3% (198/889) of all *NTRK*‐positive cases, a percentage closer to our observation with *MET* (13/122 patients, 10.7%).

One interesting observation was the high frequency of *TP53* alterations in *MET*+ LUAD, which amounted to 74.1% (20/27). In comparison, *TP53* has altered in 52.3% of (296/566) LUAD cases in The Cancer Genome Atlas database^8^ and 61.1% (151/247) LUAD cases in the MSK‐IMPACT database, respectively.^39^ The difference in incidence rates was not statistically significant for either comparison, which could be due to the small number of *MET*+ LUAD cases. This finding warrants further validation and may provide alternative therapeutic targets for *MET*+ LUAD patients. *MET* fusions are prohibitively rare for clinical trials of a *MET*‐altered cohort and as a result, no such trial has been reported to date.^1^

Our findings also provided candidates for a mechanistic study that may translate into therapeutic inventions. Of the 58 unique fusion partners identified in our study, 48 were novel. Importantly, among the corresponding fusions, 13 were in‐frame with KD‐intact *MET* as the 3' partner, involving 5' partners actionable targets such as protein kinase A (*PRKAR1A*),^40^ targets with ongoing drug development such as Tousled‐like kinase 2 (*TLK2)*,^41^ proposed prognostic biomarkers such as *LRIG* ^42^ and *DOCK4*,^43^ and genes with preclinical evidence of cancer‐associated activity such as *DIXDC1* ^44^ and *YEATS2* .^45^ Functional insights into the updated list may open avenues to new therapies for *MET*+ cancers. Also noteworthy were the fusions with *MET* as the 5' partner. The putative protein products may not undergo dimerization and subsequent MET transactivation and yet still be clinically relevant, as suggested by an interesting case report of *MET‐UBE2H* as a mechanism of acquired EGFR resistance in LC.^16^

In addition to partners, breakpoints that result in the inclusion of *MET* exon 14 may result in elevated oncogenic activity of the chimeric protein. For instance, our third highlighted case and a previously reported one^13^ shared tumor histology (LUAD), 5' partner (exons 1‐24 of *KIF5B*), and regimen, although the former lacked *MET* exon 14. Nonetheless, the two cases showed vastly different responses to crizotinib as the former had a PFS of 2 months with best response of stable disease, while the response lasted for 10 months for the patient whose *KIF5B‐MET* included exon 14. This contrast was consistent with the observation that the prognosis for patients with *MET* exon 14 skipping was generally worse compared with *MET*‐amplified NSCLC.^46^

This study was limited by several factors. First, the low prevalence of *MET* fusions and heterogeneity of partners and breakpoint require large cohort sizes for high‐confidence characterization. Second, although this study is to date the largest effort in this field, *MET*+ patients were still scarce except in LC, which limited the depth of insights gained from the remaining tumor types. Third, *MET* fusions were identified from DNA‐based genomic profiles and were not validated on the mRNA or protein level. Therefore, the putative fused genes reported in our study will need further validation on the RNA and/or protein level. Fourth, the therapeutic data of most enrolled patients were not available. Therefore, it is challenging to determine whether the *MET* fusion is a primary or acquired event. The study could benefit from more detailed clinical information, especially those regarding treatment and response/progression, which could have led to the characterization of drug efficacy for different *MET* fusions and potential resistance mechanisms of *MET*+ patients.

## 5 CONCLUSION

To our knowledge, this study is currently the largest in characterizing *MET* fusions. Our findings provide insights into the structure, partner distribution, and concurrent genomic alterations of *MET* fusions. This study warrants further clinical validation and mechanistic investigations that may translate into therapeutic avenues for *MET*+ cancer patients.

## AUTHOR CONTRIBUTIONS

**Hui Xia:** Conceptualization (equal); data curation (equal); formal analysis (equal); writing – original draft (equal); writing – review and editing (equal). **Junhua Zhang:** Conceptualization (equal); data curation (equal); formal analysis (equal); writing – original draft (equal); writing – review and editing (equal). **Tong Chen:** Data curation (equal). **Mingzhao Wang:** Data curation (equal). **Dongna Chen:** Data curation (equal). **Tongguo si:** Data curation (equal); project administration (equal); writing – review and editing (equal). **Yutao Liu:** Data curation (equal); project administration (equal); writing – review and editing (equal).

## FUNDING INFORMATION

This study was supported by the Translational research project of the Medical Oncology Key Foundation of Cancer Hospital Chinese Academy of Medical Sciences (CICAMS‐MOTRP2022002) and the Beijing Medical Award Foundation (YXJL‐2020‐0785‐1174).

## CONFLICT OF INTEREST STATEMENT

The authors have no conflict of interest to declare.

## ETHICS APPROVAL AND CONSENT TO PARTICIPATE

All procedures performed in studies involving human participants were in accordance with the ethical standards of the Institutional Review Board of Tianjin Medical University Cancer Hospital and Institute and with the 1964 Helsinki Declaration and its later amendments or comparable ethical standards.

## PATIENT CONSENT FOR PUBLICATION

All patients had provided written informed consent for participating in the study.

## Supporting information


Figure S1.

Table S1.

Table S2.

Data S1.

## Data Availability

The datasets used and/or analyzed during the current study are available from the corresponding author upon reasonable request.

## References

[1] Guo R , Luo J , Chang J , Rekhtman N , Arcila M , Drilon A . MET‐dependent solid tumours—molecular diagnosis and targeted therapy, nature reviews. Clin Oncol. 2020;17:569‐587. 10.1038/s41571-020-0377-z PMC7478851 32514147

[2] Ho‐Yen CM , Jones JL , Kermorgant S . The clinical and functional significance of c‐met in breast cancer: a review. Breast Cancer Res. 2015;17:52. 25887320 10.1186/s13058-015-0547-6 PMC4389345

[3] Friedlaender A , Drilon A , Banna GL , Peters S , Addeo A . The METeoric rise of MET in lung cancer. Cancer. 2020;126:4826‐4837. 32888330 10.1002/cncr.33159 PMC9066984

[4] Zhang M , Li G , Sun X , et al. MET amplification, expression, and exon 14 mutations in colorectal adenocarcinoma. Hum Pathol. 2018;77:108‐115. 29641976 10.1016/j.humpath.2018.03.024

[5] Fujino T , Suda K , Mitsudomi T . Emerging MET tyrosine kinase inhibitors for the treatment of non‐small cell lung cancer. Expert Opin Emerg Drugs. 2020;25:229‐249. 32615820 10.1080/14728214.2020.1791821

[6] Zhang Y , Xia M , Jin K , et al. Function of the c‐met receptor tyrosine kinase in carcinogenesis and associated therapeutic opportunities. Mol Cancer. 2018;17:45. 29455668 10.1186/s12943-018-0796-y PMC5817860

[7] Lee JK , Madison R , Classon A , et al. Characterization of non‐small‐cell Lung cancers with MET exon 14 skipping alterations detected in tissue or liquid: Clinicogenomics and real‐world treatment patterns. JCO Precis Oncol. 2021;5:1354‐1376. 10.1200/PO.21.00122 PMC8407654 34476332

[8] C.G.A.R. Network . Comprehensive molecular profiling of lung adenocarcinoma. Nature. 2014;511:543‐550. 25079552 10.1038/nature13385 PMC4231481

[9] Schrock AB , Frampton GM , Suh J , et al. Characterization of 298 patients with lung cancer harboring MET exon 14 skipping alterations. J Thorac Oncol. 2016;11:1493‐1502. 27343443 10.1016/j.jtho.2016.06.004

[10] Aisner DL , Sholl LM , Berry LD , et al. The impact of smoking and TP53 mutations in Lung adenocarcinoma patients with targetable mutations‐the Lung cancer mutation consortium (LCMC2). Clin Cancer Res. 2018;24:1038‐1047. 29217530 10.1158/1078-0432.CCR-17-2289 PMC7008001

[11] Lee HE , Kim MA , Lee HS , et al. MET in gastric carcinomas: comparison between protein expression and gene copy number and impact on clinical outcome. Br J Cancer. 2012;107:325‐333. 22644302 10.1038/bjc.2012.237 PMC3394975

[12] Pal SK , Ali SM , Yakirevich E , et al. Characterization of clinical cases of advanced papillary renal cell carcinoma via comprehensive genomic profiling. Eur Urol. 2018;73:71‐78. 28592388 10.1016/j.eururo.2017.05.033

[13] Cho JH , Ku BM , Sun J‐M , et al. KIF5B‐MET gene rearrangement with robust antitumor activity in response to crizotinib in lung adenocarcinoma. J Thorac Oncol. 2018;13:e29‐e31. 29102694 10.1016/j.jtho.2017.10.014

[14] Davies KD , Ng TL , Estrada‐Bernal A , et al. Dramatic response to crizotinib in a patient with lung cancer positive for an HLA‐DRB1‐MET gene fusion. JCO Precis Oncol. 2017;2017:1‐6. 10.1200/PO.17.00117 PMC5844276 29527595

[15] Zhu YC , Wang WX , Xu CW , et al. Identification of a novel crizotinib‐sensitive MET‐ATXN7L1 gene fusion variant in lung adenocarcinoma by next generation sequencing. Ann Oncol. 2018;29:2392‐2393. 30339198 10.1093/annonc/mdy455

[16] Zhu Y‐C , Wang W‐X , Song Z‐B , et al. MET‐UBE2H fusion as a novel mechanism of acquired EGFR resistance in lung adenocarcinoma. J Thorac Oncol. 2018;13:e202‐e204. 30244854 10.1016/j.jtho.2018.05.009

[17] Plenker D , Bertrand M , de Langen AJ , et al. Structural alterations of MET trigger response to MET kinase inhibition in Lung adenocarcinoma patients. Clin Cancer Res. 2018;24:1337‐1343. 29284707 10.1158/1078-0432.CCR-17-3001

[18] Wang Wx , Xu C , Chen Y , et al. MET gene fusions in non‐small cell lung cancer (NSCLC) in the Chinese population: a multicenter study. J Clin Oncol. 2018;36:e13539.

[19] Zhuo M , Liang Z , Yi Y , et al. Analysis of MET kinase domain rearrangement in NSCLC. Lung Cancer. 2020;145:140‐143. 32447117 10.1016/j.lungcan.2020.04.040

[20] Zhang K , Chen H , Wang Y , et al. Clinical characteristics and molecular patterns of ‐rearranged Lung cancer in Chinese patients. Oncol Res. 2019;27:575‐582. 30131091 10.3727/096504018X15344979253618 PMC7848427

[21] Li YS , Jiang BY , Yang JJ , et al. Unique genetic profiles from cerebrospinal fluid cell‐free DNA in leptomeningeal metastases of EGFR‐mutant non‐small‐cell lung cancer: a new medium of liquid biopsy. Ann Oncol. 2018;29:945‐952. 29346604 10.1093/annonc/mdy009

[22] Mao X , Zhang Z , Zheng X , et al. Capture‐based targeted Ultradeep sequencing in paired tissue and plasma samples demonstrates differential subclonal ctDNA‐releasing capability in advanced Lung cancer. J Thorac Oncol. 2017;12:663‐672. 28007624 10.1016/j.jtho.2016.11.2235

[23] Li H , Durbin R . Fast and accurate short read alignment with burrows‐wheeler transform. Bioinformatics. 2009;25:1754‐1760. 19451168 10.1093/bioinformatics/btp324 PMC2705234

[24] McKenna A , Hanna M , Banks E , et al. The genome analysis toolkit: a MapReduce framework for analyzing next‐generation DNA sequencing data. Genome Res. 2010;20:1297‐1303. 20644199 10.1101/gr.107524.110 PMC2928508

[25] Koboldt DC , Zhang Q , Larson DE , et al. VarScan 2: somatic mutation and copy number alteration discovery in cancer by exome sequencing. Genome Res. 2012;22:568‐576. 22300766 10.1101/gr.129684.111 PMC3290792

[26] Newman AM , Bratman SV , Stehr H , et al. FACTERA: a practical method for the discovery of genomic rearrangements at breakpoint resolution. Bioinformatics. 2014;30:3390‐3393. 25143292 10.1093/bioinformatics/btu549 PMC4296148

[27] Wang K , Li M , Hakonarson H . ANNOVAR: functional annotation of genetic variants from high‐throughput sequencing data. Nucleic Acids Res. 2010;38:e164. 20601685 10.1093/nar/gkq603 PMC2938201

[28] Cingolani P , Platts A , Wang LL , et al. A program for annotating and predicting the effects of single nucleotide polymorphisms, SnpEff: SNPs in the genome of Drosophila melanogaster strain w1118; iso‐2; iso‐3. Fly. 2012;6:80‐92. 22728672 10.4161/fly.19695 PMC3679285

[29] Cooper CS , Park M , Blair DG , et al. Molecular cloning of a new transforming gene from a chemically transformed human cell line. Nature. 1984;311:29‐33. 6590967 10.1038/311029a0

[30] Cheng T , Gu Z , Song D , et al. Genomic and clinical characteristics of MET exon14 alterations in a large cohort of Chinese cancer patients revealed distinct features and a novel resistance mechanism for crizotinib. J Cancer. 2021;12:644‐651. 33403024 10.7150/jca.49391 PMC7778531

[31] Liu XW , Chen XR , Rong YM , et al. MET exon 14 skipping mutation, amplification and overexpression in pulmonary sarcomatoid carcinoma: a multi‐center study. Transl Oncol. 2020;13:100868. 32920328 10.1016/j.tranon.2020.100868 PMC7492996

[32] Rivalland G , Mitchell P , Murone C , et al. Mesenchyme to epithelial transition protein expression, gene copy number and clinical outcome in a large non‐small cell lung cancer surgical cohort, Transl Lung. Cancer Res. 2019;8:167‐175. 10.21037/tlcr.2019.03.11 PMC6504649 31106127

[33] Bubendorf L , Dafni U , Schöbel M , et al. Prevalence and clinical association of MET gene overexpression and amplification in patients with NSCLC: results from the European thoracic oncology platform (ETOP) Lungscape project. Lung Cancer. 2017;111:143‐149. 28838386 10.1016/j.lungcan.2017.07.021

[34] Pekova B , Sykorova V , Dvorakova S , et al. RET, NTRK, ALK, BRAF, and MET fusions in a large cohort of pediatric papillary thyroid carcinomas. Thyroid. 2020;30:1771‐1780. 32495721 10.1089/thy.2019.0802

[35] Yang W , Zhao X , Zheng A , et al. Identification of MET fusions in solid tumors: a multicenter, large scale study in China. Int J Cancer. 2023;152:1259‐1268. 36408924 10.1002/ijc.34361

[36] Recurrent MET fusion genes represent a drug target in pediatric glioblastoma. Nat Med. 2016;22:1314‐1320. 27748748 10.1038/nm.4204

[37] Bao Z‐S , Chen H‐M , Yang M‐Y , et al. RNA‐seq of 272 gliomas revealed a novel, recurrent PTPRZ1‐MET fusion transcript in secondary glioblastomas. Genome Res. 2014;24:1765‐1773. 25135958 10.1101/gr.165126.113 PMC4216918

[38] Noh K‐W , Lee M‐S , Lee SE , et al. Molecular breakdown: a comprehensive view of anaplastic lymphoma kinase (ALK)‐rearranged non‐small cell lung cancer. J Pathol. 2017;243:307‐319. 28741662 10.1002/path.4950

[39] Zehir A , Benayed R , Shah RH , et al. Mutational landscape of metastatic cancer revealed from prospective clinical sequencing of 10,000 patients. Nat Med. 2017;23:703‐713. 28481359 10.1038/nm.4333 PMC5461196

[40] Coles GL , Cristea S , Webber JT , et al. Unbiased proteomic profiling uncovers a targetable GNAS/PKA/PP2A Axis in small cell Lung cancer stem cells. Cancer Cell. 2020;38:129‐143.e7. 32531271 10.1016/j.ccell.2020.05.003 PMC7363571

[41] Lee S‐B , Chang T‐Y , Lee N‐Z , Yu Z‐Y , Liu C‐Y , Lee H‐Y . Design, synthesis and biological evaluation of bisindole derivatives as anticancer agents against tousled‐like kinases. Eur J Med Chem. 2021;227:113904. 34662748 10.1016/j.ejmech.2021.113904

[42] Kvarnbrink S , Karlsson T , Edlund K , et al. LRIG1 is a prognostic biomarker in non‐small cell lung cancer. Acta Oncol. 2015;54:1113‐1119. 25813475 10.3109/0284186X.2015.1021427

[43] Westbrook JA , Wood SL , Cairns DA , et al. Identification and validation of DOCK4 as a potential biomarker for risk of bone metastasis development in patients with early breast cancer. J Pathol. 2019;247:381‐391. 30426503 10.1002/path.5197 PMC6618075

[44] Xu Z , Liu D , Fan C , Luan L , Zhang X , Wang E . DIXDC1 increases the invasion and migration ability of non‐small‐cell lung cancer cells via the PI3K‐AKT/AP‐1 pathway. Mol Carcinog. 2014;53:917‐925. 23813858 10.1002/mc.22059

[45] Mi W , Guan H , Lyu J , et al. YEATS2 links histone acetylation to tumorigenesis of non‐small cell lung cancer. Nat Commun. 2017;8:1088. 29057918 10.1038/s41467-017-01173-4 PMC5651844

[46] Tong JH , Yeung SF , Chan AWH , et al. MET amplification and exon 14 splice site mutation define unique molecular subgroups of non‐small cell Lung carcinoma with poor prognosis. Clin Cancer Res. 2016;22:3048‐3056. 26847053 10.1158/1078-0432.CCR-15-2061

