# Supplementary figures and images for: Molecular characterization of MET fusions from a large real‐world Chinese population: A multicenter study
Source: Cancer Med. 2023 Jun 16;12(13):14015–24. doi: 10.1002/cam4.6047 (PMC10358190; doi:10.1002/cam4.6047)

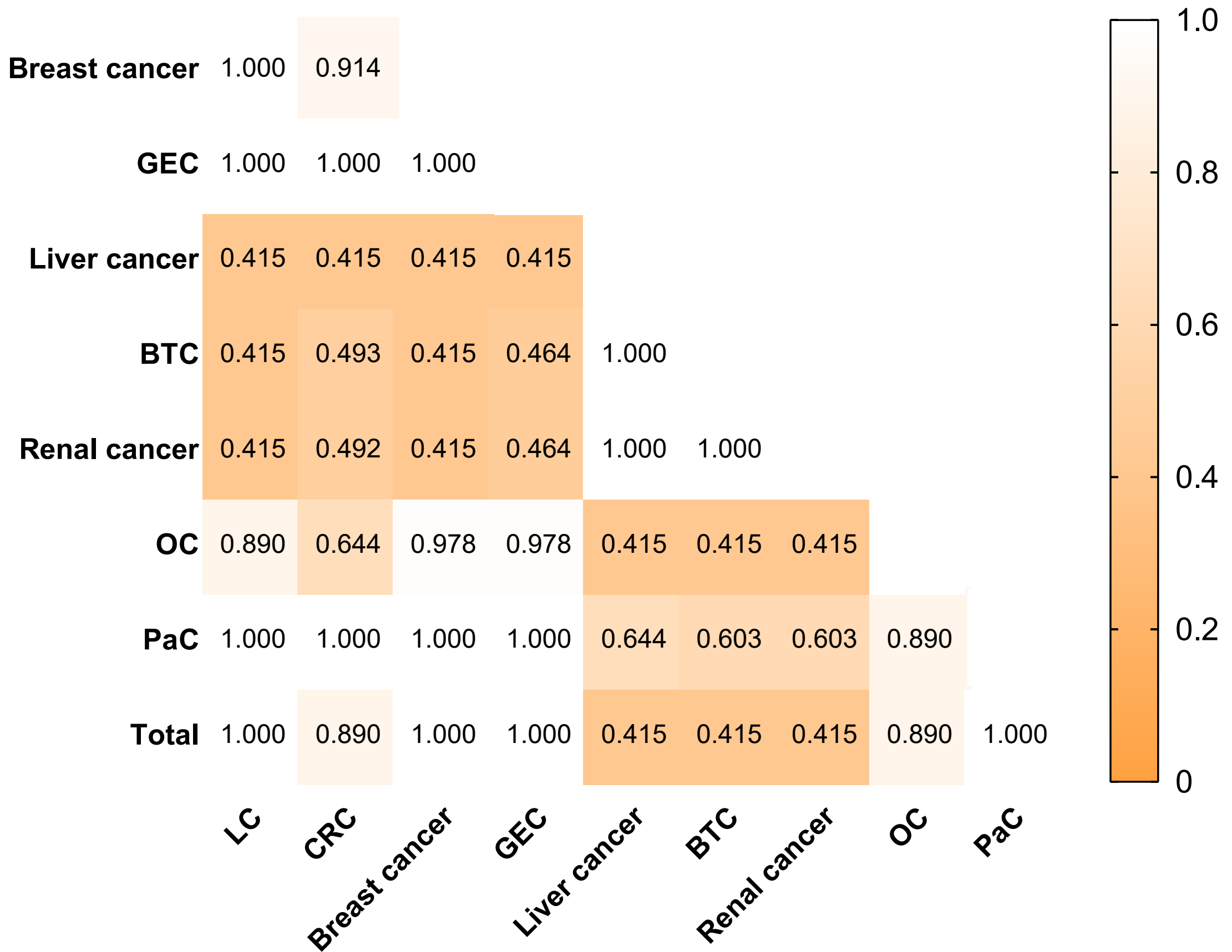

Supplement: Supplementary file 1 — Figure S1. [file CAM4-12-14015-s004.pdf]
