# Supplementary material for: Molecular characterization of MET fusions from a large real‐world Chinese population: A multicenter study
Source: Cancer Med. 2023 Jun 16;12(13):14015–24. doi: 10.1002/cam4.6047 (PMC10358190; doi:10.1002/cam4.6047)
Supplement: Supplementary file 2 — Table S1. [file CAM4-12-14015-s003.docx]

| **Table S1. Clinical and molecular characteristics for each *MET* fusion-positive patient.** | | | | | | | | |  |  |  |  |  |  |
| --- | --- | --- | --- | --- | --- | --- | --- | --- | --- | --- | --- | --- | --- | --- |
| **Patient No.** | ***MET* Fusion** | **Cancer type** | **Stage** | **Sex** | **Age** | **Sample type** | **Number of targeted genes** | **Intergenic fusion?** | **Novel partner?** | **Has MET**  **kinase domain?** | **Has *MET* exon 14?** | **In-frame?** | ***MET* as 3' partner?** | **Fusion partner with known dimerization domain?** |
| p1 | intergenic(*ANKRD7,LINC02476*)-*MET* | LC | IV | M | 67 | Plasma | 168 | yes |  | yes | no |  | yes |  |
| p2 | *TFEC-MET* | PaC | IV | M | 50 | Tissue | 168 | no | yes | yes | no |  | yes | no |
| p3 | *MET*-intergenic(*LINC01510,MET*) | LC | IV | F | 48 | Plasma | 168 | yes |  | no | no |  | no |  |
|  | *MET*-intergenic(*LOC102724434,CAV2*) | LC | IV | F | 48 | Plasma | 168 | yes |  | no | no |  | no |  |
|  | intergenic(*LINC01392,SNORA25B*)-*MET* | LC | IV | F | 48 | Plasma | 168 | yes |  | yes | no |  | yes |  |
|  | *FOXP2-MET* | LC | IV | F | 48 | Plasma | 168 | no | yes | yes | no |  | yes | no |
|  | *MET*-intergenic(*TFEC,TES*) | LC | IV | F | 48 | Plasma | 168 | yes |  | no | yes |  | no |  |
| p4 | *TPRXL-MET* | CRC | IV | F | 43 | Tissue | 41 | no | yes | yes | yes |  | yes | no |
| p5 | *MET*-intergenic(*CAPZA2,ST7-AS1*) | GEC | II | M | 72 | Plasma | 520 | yes |  | no | no |  | no |  |
| p6 | intergenic(*LINC01510,MET*)-*MET* | LC | II | NA | NA | Tissue | 520 | yes |  | yes | yes |  | yes |  |
|  | *MET-DOCK4* | LC | II | NA | NA | Tissue | 520 | no | yes | no | no |  | no |  |
| p7 | *CAPZA2-MET* | LC | III | M | 61 | Plasma | 520 | no | no | yes | yes |  | yes | no |
| p8 | *MET-HLA-DRB5* | LC | II | M | 48 | Tissue | 520 | no | yes | no | no |  | no |  |
| p9 | *MIER1-MET* | LC | IV | M | 57 | Tissue | 8 | no | yes | yes | no |  | yes | no |
|  | *HLA-DRB1-MET* | LC | IV | M | 57 | Tissue | 8 | no | no | yes | no | yes | yes | no |
| p10 | *DPP6-MET* | CRC | III | M | 57 | Plasma | 168 | no | yes | yes | yes |  | yes | no |
| p11 | *MET*-intergenic(*LOC100129620,PLPPR4*) | LC | III | F | 63 | Plasma | 168 | yes |  | no | yes |  | no |  |
| p12 | intergenic(*LINC02476,KCND2*)-*MET* | LC | III | F | 44 | Tissue | 8 | yes |  | yes | yes |  | yes |  |
| p13 | *TLK2-MET* | LC | IV | F | 65 | Plasma | 168 | no | yes | yes | yes | yes | yes | no |
| p13 | *MET-DOCK4* | LC | IV | F | 65 | Plasma | 168 | no | yes | no | no |  | no |  |
| p14 | *GJC2-MET* | LC | III | M | 49 | Tissue | 520 | no | yes | yes | yes |  | yes | no |
| p15 | *MET-IFRD1* | LC | III | F | 63 | NA | 520 | no | yes | no | no |  | no |  |
| p15 | *MET*-intergenic(*LOC102724434,CAV2*) | LC | III | F | 63 | NA | 520 | yes |  | no | yes |  | no |  |
| p16 | *TFEC-MET* | LC | III | F | 70 | NA | 168 | no | yes | yes | yes | yes | yes | no |
| p17 | *LOC349160-MET* | LC | III | F | 60 | Plasma | 168 | no | yes | yes | yes |  | yes |  |
| p18 | intergenic(*IRS2,LINC00396*)-*MET* | LC | III | M | 52 | Plasma | 168 | yes |  | yes | yes |  | yes |  |
| p19 | intergenic(*ANKRD7,LINC02476*)-*MET* | LC | III | M | 46 | Plasma | 168 | yes |  | no | no |  | yes |  |
| p20 | *SPAST-MET* | LC | III | M | 58 | Plasma | 168 | no | yes | no | no |  | yes | no |
| p21 | *ST7-MET* | LC | III | M | 87 | Tissue | 520 | no | no | no | no | yes | yes | no |
| p22 | *MET-FOXP2* | LC | III | M | 57 | Tissue | 68 | no | yes | no | no |  | no |  |
| p23 | *MET-CFTR* | LC | III | F | 77 | Plasma | 168 | no | yes | no | yes |  | no |  |
| p24 | *CADPS2-MET* | LC | III | F | 54 | Plasma | 520 | no | yes | yes | yes | yes | yes | no |
| p25 | intergenic (*ZNF621,CTNNB1*)-*MET* | LC | III | M | 61 | Plasma | 520 | yes |  | yes | yes |  | yes |  |
| p26 | *MET*-intergenic(*MET,CAPZA2*) | LC | III | F | 56 | PE | 168 | yes |  | no | no |  | no |  |
| p27 | *CFTR-MET* | LC | I | M | 57 | Plasma | 168 | no | yes | yes | yes |  | yes | no |
| p28 | *KIF5B-MET* | LC | III | M | 56 | Tissue | 168 | no | no | yes | no | yes | yes | yes |
| p29 | *HLA-DRB1-MET* | LC | III | F | 65 | PE | 8 | no | no | yes | yes |  | yes | no |
| p30 | *MET-BBX* | LC | III | M | 79 | Tissue | 168 | no | yes | no | yes |  | no |  |
| p31 | intergenic(*MET,CAPZA2*)-*ING3* | LC | III | F | 47 | Tissue | 520 | yes |  | no | no |  | no |  |
| p32 | intergenic(*CAPZA2,ST7-AS1*)-*MET* | LC | III | M | 60 | NA | 168 | yes |  | yes | yes |  | yes |  |
| p33 | *LINC01510-MET* | LC | III | M | 66 | Tissue | 520 | no | yes | yes | yes |  | yes |  |
| p34 | intergenic(*IGFL4,IGFL3*)-*MET* | LC | III | M | 82 | Tissue | 8 | yes |  | yes | yes |  | yes |  |
| p35 | intergenic(*LINC00174,GS1-124K5.4*)-*MET* | LC | III | M | 56 | CSF | 168 | yes |  | yes | yes |  | yes |  |
| p36 | *MET-LINC01392* | LC | III | F | 63 | Plasma | 168 | no | yes | no | no |  | no |  |
| p37 | intergenic(*LINC01446,LINC01445*)-*MET* | Others | NA | M | 58 | NA | 520 | yes |  | yes | yes |  | yes |  |
|  | *MET*-intergenic(*OR6B1,OR2A5*) | Others | NA | M | 58 | NA | 520 | yes |  | no | no |  | no |  |
| p38 | *MET*-intergenic(*LSM8,ANKRD7*) | LC | III | F | 75 | PE | 520 | yes |  | no | no |  | no |  |
| p39 | *CAPZA2-MET* | LC | III | M | 52 | PE | 520 | no | no | yes | yes |  | yes | no |
| p40 | *DOCK4-MET* | LC | III | NA | NA | Tissue | 168 | no | yes | no | no | yes | yes | no |
| p41 | *MET-PLEKHA5* | LC | NA | M | 65 | Tissue | 520 | no | yes | no | no |  | no |  |
| p42 | *MET*-intergenic(*HLA-DRB6,HLA-DRB1*) | LC | III | F | 51 | Tissue | 520 | yes |  | no | no |  | no |  |
| p43 | *MET-CFTR* | GEC | III | M | 63 | Plasma | 520 | no | yes | no | no |  | no |  |
| p44 | *CAV1-MET* | CRC | III | F | 45 | Tissue | 520 | no | no | no | no |  | yes | no |
| p45 | *SRPK1-MET* | LC | I | F | 53 | Tissue | 520 | no | yes | yes | yes |  | yes | no |
| p46 | *MET-ST7* | LC | III | M | 66 | Tissue | 68 | no | no | no | yes |  | no |  |
| p47 | *MET*-intergenic(*FOXP2,MDFIC*) | LC | III | M | 64 | Tissue | 168 | yes |  | no | yes |  | no |  |
| p48 | *ASXL1-MET* | CRC | III | M | 57 | Tissue | 520 | no | yes | no | no |  | yes | no |
| p49 | *MGAT5B-MET* | LC | III | F | 54 | PE | 68 | no | yes | yes | yes |  | yes | no |
| p50 | *MET-STEAP4* | LC | III | F | 42 | Tissue | 168 | no | yes | no | no |  | no |  |
| p51 | intergenic(*MYC,PVT1*)*-MET* | BTC | III | F | 42 | Plasma | 520 | yes |  | yes | yes |  | yes |  |
| p52 | *MET*-intergenic(*SMPDL3A,CLVS2*) | LC | III | M | 64 | Tissue | 520 | yes |  | no | no |  | no |  |
| p53 | intergenic(*METTL2B,LINC01000*)-*MET* | BRCA | NA | NA | NA | Tissue | 520 | yes |  | yes | yes |  | yes |  |
| p54 | intergenic(*ANKRD7,LINC02476*)-*MET* | LC | III | F | 49 | Plasma | 168 | yes |  | yes | yes |  | yes |  |
| p55 | *HLA-DRB1-MET* | LC | III | M | 68 | Plasma | 168 | no | no | yes | no | yes | yes | no |
| p56 | *MET*-intergenic(*MET,CAPZA2*) | LC | III | F | 50 | Tissue | 168 | yes |  | no | yes |  | no |  |
| p57 | *CAV1-MET* | LC | II | M | 67 | Tissue | 520 | no | no | yes | yes | yes | yes | no |
| p58 | *CAPZA2-MET* | LC | III | F | 69 | Plasma | 168 | no | no | no | no |  | yes | no |
| p59 | *MDFIC-MET* | LC | III | M | 40 | Tissue | 168 | no | yes | yes | yes |  | yes | no |
| p60 | *MET*-intergenic(*MET,CAPZA2*) | LC | III | M | 68 | Plasma | 520 | yes |  | no | no |  | no |  |
| p61 | *MET-ADAP1* | LC | III | F | 48 | Plasma | 168 | no | yes | no | no |  | no |  |
| p62 | *MET*-intergenic(*EZH2,RNY5*) | LC | III | M | 70 | Plasma | 168 | yes |  | no | yes |  | no |  |
| p63 | intergenic(*LOC102724434,CAV2*)-*MET* | LC | III | M | 47 | Plasma | 168 | yes |  | yes | yes |  | yes |  |
| p64 | *LAMB1-MET* | LC | I | F | 52 | Tissue | 520 | no | yes | yes | no | yes | yes | no |
| p65 | *COG5-MET* | LC | NA | M | 27 | Plasma | 168 | no | yes | yes | yes |  | yes | no |
| p66 | *PRKAR1A-MET* | LC | IV | M | 74 | Plasma | 168 | no | yes | yes | no | yes | yes | no |
| p67 | *MET*-intergenic(*LOC107986794,POM121L12*) | LC | III | M | 52 | Plasma | 8 | yes |  | no | no |  | no |  |
| p68 | *AKAP9-MET* | LC | IV | M | 79 | NA | 168 | no | yes | yes | yes |  | yes | no |
| p69 | *HLA-DRB1-MET* | LC | IV | F | 67 | Plasma | 520 | no | no | yes | no | yes | yes | no |
| p70 | *MET*-intergenic(*LINC01392,SNORA25B*) | LC | IV | M | 60 | Plasma | 168 | yes |  | no | no |  | no |  |
| p71 | intergenic(*NKX2-4,NKX2-2*)-*MET* | LC | III | F | 59 | Tissue | 8 | yes |  | yes | yes |  | yes |  |
| p72 | *MET*-intergenic(*LINC02494,LINC02429*) | LC | IV | M | 61 | PE | 8 | yes |  | no | yes |  | no |  |
| p73 | *MET*-intergenic(*ASZ1,CFTR*) | LC | III | M | 59 | Tissue | 168 | yes |  | no | yes |  | no |  |
| p74 | intergenic(*CALCR,MIR4652*)-*MET* | LC | IV | M | 69 | Plasma | 520 | yes |  | yes | yes |  | yes |  |
|  | *MET*-intergenic(*CACNA2D1,PCLO*) | LC | IV | M | 69 | Plasma | 520 | yes |  | no | yes |  | no |  |
| p75 | *KCND2-MET* | LC | IV | F | 57 | Plasma | 168 | no | yes | yes | yes |  | yes | no |
| p76 | *MET*-intergenic(*LINC01510,MET*) | LC | IV | M | 55 | Tissue | 8 | yes |  | no | no |  | no |  |
| p77 | *ST7-MET* | Others | IV | M | 61 | Tissue | 520 | no | no | yes | no | yes | yes | no |
| p78 | *ECT2-MET* | LC | IV | M | 66 | Tissue | 8 | no | yes | no | no |  | yes | no |
| p79 | intergenic(*TFEC,TES*)-*MET* | CRC | IV | F | 55 | Tissue | 41 | yes |  | yes | yes |  | yes |  |
|  | *MET*-intergenic(*TFEC,TES*) | CRC | IV | F | 55 | Tissue | 41 | yes |  | no | no |  | no |  |
| p80 | *CD47-MET* | LC | NA | NA | NA | PE | 520 | no | no | yes | no | yes | yes | no |
| p80 | *WEE2-MET* | LC | NA | NA | NA | PE | 520 | no | yes | yes | no |  | yes | no |
| p81 | *GTF2IRD1-MET* | LICA | IV | M | 62 | Tissue | 520 | no | yes | yes | no | yes | yes | no |
| p82 | *EML4-MET* | CRC | IV | M | 61 | Plasma | 520 | no | yes | yes | no | yes | yes | no |
| p83 | intergenic(*LINC01392,SNORA25B*)-*MET* | BRCA | IV | F | 58 | Tissue | 520 | yes |  | yes | yes |  | yes |  |
| p84 | *MET*-intergenic(*LINC01572,ZFHX3*) | LC | IV | M | 65 | Plasma | 168 | yes |  | no | no |  | no |  |
|  | *MET*-intergenic(*FAM3C,PTPRZ1*) | LC | IV | M | 65 | Plasma | 168 | yes |  | no | no |  | no |  |
|  | *TRIM4-MET* | LC | IV | M | 65 | Plasma | 168 | no | no | yes | no | yes | yes | yes |
|  | intergenic(*FAM3C,PTPRZ1*)-*MET* | LC | IV | M | 65 | Plasma | 168 | yes |  | yes | yes |  | yes |  |
|  | *MET*-intergenic(*FAM3C,PTPRZ1*) | LC | IV | M | 65 | Plasma | 168 | yes |  | no | no |  | no |  |
| p85 | *DIXDC1-MET* | LICA | III | F | 50 | Tissue | 520 | no | yes | yes | no | yes | yes | no |
| p86 | *MET*-intergenic(*MET,CAPZA2*) | LC | IV | F | 49 | Tissue | 520 | yes |  | no | yes |  | no |  |
| p87 | intergenic(*STRA8,CNOT4*)-*MET* | CRC | IV | NA | NA | Tissue | 520 | yes |  | yes | yes |  | yes |  |
|  | *CHL1-MET* | CRC | IV | NA | NA | Tissue | 520 | no | yes | yes | yes |  | yes | no |
| p88 | *MET-KCND2* | LC | IV | M | 53 | Tissue | 520 | no | yes | no | yes |  | no |  |
|  | *AASS-MET* | LC | IV | M | 53 | Tissue | 520 | no | yes | yes | yes |  | yes | no |
|  | *MET*-intergenic(*FAM3C,PTPRZ1*) | LC | IV | M | 53 | Tissue | 520 | yes |  | no | no |  | no |  |
| p89 | *THAP5-MET* | LC | III | M | 68 | Tissue | 168 | no | yes | yes | yes |  | yes | no |
| p90 | intergenic(*ZNF716,ZNF733P*)-*MET* | LC | IV | M | NA | Tissue | 520 | yes |  | yes | yes |  | yes |  |
| p91 | *LRIG3-MET* | LC | IV | F | 52 | Tissue | 520 | no | yes | yes | no | yes | yes | no |
| p92 | *KIF5B-MET* | LC | III | F | 46 | NA | 520 | no | no | yes | no | yes | yes | yes |
|  | *CDR2-MET* | LC | III | F | 46 | NA | 520 | no | yes | yes | no |  | yes | no |
| p93 | *CAPZA2-MET* | LC | III | M | 58 | Plasma | 168 | no | no | yes | yes |  | yes | no |
| p94 | *MET-KIF5B* | LC | IV | F | 50 | NA | 520 | no | no | no | yes |  | no | yes |
|  | *KIF5B-MET* | LC | IV | F | 50 | NA | 520 | no | no | yes | no | yes | yes | yes |
| p95 | *GOLGA4-MET* | BTC | IV | F | 50 | Tissue | 520 | no | yes | yes | no | yes | yes | no |
|  | *KAT2B-MET* | BTC | IV | F | 50 | Tissue | 520 | no | yes | yes | no |  | yes | no |
| p96 | *DLGAP1-MET* | LC | IV | F | 48 | PE | 168 | no | yes | yes | yes |  | yes | no |
| p97 | intergenic(*SNORA25B,TFEC*)-*MET* | LICA | III | F | 80 | Plasma | 520 | yes |  | yes | yes |  | yes |  |
| p98 | *MET*-intergenic(*LSM8,ANKRD7*) | LC | III | M | 44 | Plasma | 168 | yes |  | no | no |  | no |  |
| p99 | *MET-ARL1* | LC | II | F | 60 | Tissue | 68 | no | yes | no | no |  | no |  |
| p100 | *TFG-MET* | RECA | IV | M | 50 | Tissue | 520 | no | no | yes | no | yes | yes | yes |
| p101 | *GTF2IRD1-MET* | CRC | NA | F | 61 | Plasma | 108 | no | yes | yes | yes |  | yes | no |
| p102 | *CD47-MET* | LC | IV | M | 71 | Tissue | 520 | no | no | yes | no | yes | yes | no |
|  | *YEATS2-MET* | LC | IV | M | 71 | Tissue | 520 | no | yes | yes | yes | yes | yes | no |
| p103 | *MET*-intergenic(*C7orf66,EIF3IP1*) | LC | IV | F | 52 | Plasma | 168 | yes |  | no | no |  | no |  |
|  | *MET-FOXP2* | LC | IV | F | 52 | Plasma | 168 | no | yes | no | no |  | no |  |
| p104 | *CFTR-MET* | BRCA | IV | F | 57 | Plasma | 108 | no | yes | yes | yes | yes | yes | no |
| p105 | *MET*-intergenic(*SNORA25B,TFEC*) | CRC | NA | M | 68 | Plasma | 108 | yes |  | no | no |  | no |  |
|  | *ST7-MET* | CRC | NA | M | 68 | Plasma | 108 | no | no | no | no |  | yes | no |
| p106 | *WNT2-MET* | LC | IV | M | 70 | Tissue | 168 | no | no | no | no |  | yes | no |
|  | *POT1-MET* | LC | IV | M | 70 | Tissue | 168 | no | yes | no | no |  | yes | no |
| p107 | *KIF5B-MET* | LC | III | F | 62 | Tissue | 520 | no | no | yes | no | yes | yes | yes |
| p108 | *MET*-intergenic(*LINC01510,MET*) | CRC | NA | M | 78 | Plasma | 108 | yes |  | no | no |  | no |  |
|  | intergenic(*SNORD28B,ARID1B*)-*MET* | CRC | NA | M | 78 | Plasma | 108 | yes |  | yes | yes |  | yes |  |
| p109 | *MET*-intergenic(*LINC01392,SNORA25B*) | LC | IV | F | 54 | Plasma | 168 | yes |  | no | yes |  | no |  |
|  | intergenic(*SNORA25B,TFEC*)-*MET* | LC | IV | F | 54 | Plasma | 168 | yes |  | yes | yes |  | yes |  |
|  | *MET-ST7* | LC | IV | F | 54 | Plasma | 168 | no | no | no | yes |  | no |  |
|  | intergenic(*LINC01510,MET*)-*MET* | LC | IV | F | 54 | Plasma | 168 | yes |  | yes | yes |  | yes |  |
| p110 | *MET*-intergenic(*LINC01392,SNORA25B*) | GEC | III | M | 50 | Plasma | 520 | yes |  | no | no |  | no |  |
| p111 | *MET*-intergenic(*LOC101928211,GPR37*) | LC | III | M | 40 | Tissue | 8 | yes |  | no | no |  | no |  |
| p112 | *MET-LINC01392* | LC | III | F | 68 | Plasma | 168 | no | yes | no | yes |  | no |  |
|  | *MET*-intergenic(*TFEC,TES*) | LC | III | F | 68 | Plasma | 168 | yes |  | no | no |  | no |  |
| p113 | *LINC01392-MET* | LC | III | F | 49 | Plasma | 168 | no | yes | no | no |  | yes |  |
|  | *IMMP2L-MET* | LC | III | F | 49 | Plasma | 168 | no | yes | yes | yes |  | yes | no |
| p114 | *ST7-MET* | LC | IV | NA | NA | Tissue | 520 | no | no | yes | no | yes | yes | no |
|  | *HLA-DRB1-MET* | LC | IV | NA | NA | Tissue | 520 | no | no | yes | no | yes | yes | no |
| p115 | *CCDC178-MET* | CRC | IV | F | 65 | Tissue | 520 | no | yes | yes | no |  | yes | no |
| p116 | intergenic(*MET,CAPZA2*)-*MET* | RECA | IV | M | 59 | Tissue | 520 | yes |  | yes | yes |  | yes |  |
| p117 | *PRKAR1A-MET* | LC | III | F | 60 | Tissue | 520 | no | yes | yes | no | yes | yes | no |
| p118 | *ST7-MET* | OC | IV | F | 54 | Plasma | 520 | no | no | yes | no |  | yes | no |
| p119 | *MET-CTTNBP2* | Others | III | F | 47 | NA | 520 | no | yes | no | no |  | no |  |
| p120 | intergenic(*TFEC,TES*)-*MET* | Others | III | F | 69 | Plasma | 520 | yes |  | yes | yes |  | yes |  |
| p121 | *MAGI2-MET* | LC | IV | M | 63 | Plasma | 520 | no | yes | yes | yes |  | yes | no |
| p122 | intergenic(*LINC01392,SNORA25B*)-*MET* | LC | IV | F | 62 | Plasma | 520 | yes |  | no | no |  | yes |  |
| BTC, biliary tract cancer. CRC, colorectal cancer. GEC, gastroesophageal cancer. LC, lung cancer. OC, ovarian cancer. PaC, pancreatic cancer. NA, not available;  BRCA, breast cancer; LICA, liver cancer; RECA, renal cancer; PE, pleural effusion; CSF, Cerebrospinal fluid. | | | | | | | | | | | |  |  |  |
