# Supplementary material for: Molecular characterization of MET fusions from a large real‐world Chinese population: A multicenter study
Source: Cancer Med. 2023 Jun 16;12(13):14015–24. doi: 10.1002/cam4.6047 (PMC10358190; doi:10.1002/cam4.6047)
Supplement: Supplementary file 3 — Table S2. [file CAM4-12-14015-s001.docx]

**Table S2 *MET* fusion concurrent with *MET* amplification**

| **Patient No.** | ***MET* fusion** | **Allelic frequency** | **Concurrent *MET* amp** | ***MET* CN** |
| --- | --- | --- | --- | --- |
| p1 | intergenic(*ANKRD7,LINC02476*)-*MET* | 0.15% | Yes | 2.4 |
| p3 | *MET*-intergenic(*LINC01510,MET*) | 0.12% | Yes | 4.3 |
|  | *MET*-intergenic(*LOC102724434,CAV2*) | 0.10% |  |  |
|  | intergenic(*LINC01392,SNORA25B*)-*MET* | 0.35% |  |  |
|  | *FOXP2-MET* | 0.61% |  |  |
|  | *MET*-intergenic(*TFEC,TES*) | 0.45% |  |  |
| p5 | *MET*-intergenic(CAPZA2,ST7-AS1) | 0.35% | Yes | 2.8 |
| p13 | *TLK2-MET* | 1.53% | Yes | 2.7 |
|  | *MET-DOCK4* | 3.70% |  |  |
| p15 | *MET-IFRD1* | 3.19% | Yes | 9.2 |
|  | *MET*-intergenic(LOC102724434,CAV2) | 3.06% |  |  |
| p16 | *TFEC-MET* | 3.95% | Yes | 5.6 |
| p17 | *LOC349160-MET* | 0.10% | Yes | 2.5 |
| p18 | intergenic(*IRS2,LINC00396*)-*MET* | 3.08% | Yes | 4.3 |
| p19 | intergenic(*ANKRD7,LINC02476*)-*MET* | 0.76% | Yes | 2.5 |
| p20 | *SPAST-MET* | 4.81% | Yes | 2.5 |
| p22 | *MET-FOXP2* | 31.47% | Yes | 6.2 |
| p23 | *MET-CFTR* | 34.45% | Yes | 3 |
| p24 | *CADPS2-MET* | 1.14% | Yes | 3.9 |
| p26 | MET-intergenic(*MET,CAPZA2*) | 3.47% | Yes | 4.8 |
| p31 | intergenic(*MET,CAPZA2*)-*ING3* | 23.93% | Yes | 14.9 |
| p34 | intergenic(*IGFL4,IGFL3*)-*MET* | 1.80% | Yes | 3.3 |
| p40 | *DOCK4-MET* | 29.29% | Yes | 3.3 |
| p41 | *MET-PLEKHA5* | 8.06% | Yes | 7.8 |
| p43 | *MET-CFTR* | 6.77% | Yes | 5.6 |
| p44 | *CAV1-MET* | 45.10% | Yes | 3.7 |
| p47 | *MET*-intergenic(*FOXP2,MDFIC*) | 32.92% | Yes | 9 |
| p50 | *MET-STEAP4* | 5.46% | Yes | 3.7 |
| p51 | intergenic(*MYC,PVT1*)-*MET* | 0.66% | Yes | 3.4 |
| p54 | intergenic(*ANKRD7,LINC02476*)-*MET* | 34.38% | Yes | 3.4 |
| p59 | *MDFIC-MET* | 45.29% | Yes | 4.8 |
| p63 | intergenic(*LOC102724434,CAV2*)-*MET* | 0.50% | Yes | 3.6 |
| p65 | *COG5-MET* | 0.30% | Yes | 2.41 |
| p70 | *LINC01392-MET* | 1.01% | Yes | 4.38 |
| p74 | *CACNA2D1-MET* | 2.34% | Yes | 3.18 |
|  | *MIR4652-MET* | 1.77% |  |  |
| p75 | *KCND2-MET* | 1.61% | Yes | 2.3 |
| p77 | *ST7-AS2-MET* | 5.37% | Yes | 3.51 |
| p79 | *TES-MET* | 6.14% | Yes | 18.5 |
| p81 | *GTF2IRD1-MET* | 66.47% | Yes | 4.42 |
| p82 | *EML4-MET* | 0.72% | Yes | 2.99 |
| p83 | *LINC01392-MET* | 13.78% | Yes | 3.09 |
| p84 | *LINC01572-MET* | 0.17% | Yes | 5.23 |
| p86 | *CAPZA2-MET* | 12.69% | Yes | 29.39 |
| p88 | *PTPRZ1-MET* | 2.00% | Yes | 19.1 |
| p96 | *DLGAP1-MET* | 1.54% | Yes | 8 |
| p97 | *TFEC-MET* | 1.61% | Yes | 12.91 |
| p101 | *GTF2IRD1-MET* | 0.96% | Yes | 7.76 |
| p103 | *FLJ00325-MET* | 0.99% | Yes | 4.05 |
| p105 | *TFEC-MET* | 14.24% | Yes | 3.34 |
| p106 | *CADPS2-MET* | 2.43% | Yes | 8.91 |
|  | *POT1-MET* | 2.32% |  |  |
| p108 | *LINC01510-MET* | 1.51% | Yes | 5.4 |
|  | *CFTR-MET* | 0.94% |  |  |
| p109 | *LINC01392-MET* | 4.21% | Yes | 5.7 |
|  | *TFEC-MET* | 0.60% |  |  |
| p110 | *LINC01392-MET* | 1.31% | Yes | 3.1 |
| p111 | *LOC101928211-MET* | 3.88% | Yes | 2.9 |
| p113 | *LINC01392-MET* | 0.75% | Yes | 2.4 |
| p119 | *CTTNBP2-MET* | 13.17% | Yes | 3.6 |

CN, copy number; amp, amplification.
