# Supplementary material for: Molecular characterization of MET fusions from a large real‐world Chinese population: A multicenter study
Source: Cancer Med. 2023 Jun 16;12(13):14015–24. doi: 10.1002/cam4.6047 (PMC10358190; doi:10.1002/cam4.6047)
Supplement: Supplementary file 4 — Data S1. [file CAM4-12-14015-s002.docx]

**Supplementary figure legends**

Figure S1. Adjusted p-values of pairwise Fisher’s exact tests comparing the prevalence of *MET* fusions in the cancer types indicated in the row and column headers. BTC, biliary tract cancer. CRC, colorectal cancer. GEC, gastroesophageal cancer. LC, lung cancer. MET+, MET fusion-positive. OC, ovarian cancer. PaC, pancreatic cancer.
